# Metagenomic profiling of biliary microbiota reveals distinct microbial and functional features in cholelithiasis and cholecystic polyps

**DOI:** 10.1097/MD.0000000000049251

**Published:** 2026-06-12

**Authors:** Yuanquan Yu, Chenjie Wang, Xiang Pan, Chao Ding, Jianwei Chen

**Affiliations:** a Department of Surgery, The Second Affiliated Hospital Zhejiang University School of Medicine, Hangzhou, Zhejiang, China; b College of Pharmaceutical Science & Zhejiang-Egypt Joint Laboratory on Intelligent Discovery of Marine Drugs, Zhejiang University of Technology, Hangzhou, Zhejiang, China.

**Keywords:** bile, biliary microbiota, cholecystic polyps, cholelithiasis, metagenomic

## Abstract

Cholelithiasis and cholecystic polyps are common gastrointestinal conditions, and recent studies suggest that biliary microbiota dysbiosis may be closely associated with their pathogenesis. In this small cohort (n = 11), bile samples were aseptically collected during surgery from 6 patients with cholelithiasis and 5 patients with cholecystic polyps. Metagenomic sequencing was performed to investigate differences in the microbial composition and functional profiles between the 2 groups. The results revealed that the microbial α diversity of bile from patients with cholelithiasis was significantly greater than that of the polyp group, with significant differences in the Richness, Chao1, ACE, and Shannon indices (*P* < .05). β-diversity analysis further revealed distinct differences in microbial community composition across the groups. Linear discriminant analysis effect size analysis revealed Pseudomonadota as the only phylum enriched in the polyp group, whereas the cholelithiasis group was enriched with multiple phyla, such as Campylobacterota, Bacillota, and Fusobacteriota, and 35 genera, such as *Bacteroides*, *Mucilaginibacter*, and *Pedobacter*. Kyoto Encyclopedia of Genes and Genomes functional enrichment analysis indicated that the microbial community in the cholelithiasis group was significantly associated with neurodegenerative disease-related pathways, while the microbial community in the polyp group was enriched in pathways related to ribosomes and fluid shear stress. This study highlights the potential role of biliary microecological imbalances in the development of biliary diseases and provides a theoretical basis for exploring pathogenesis and microbiota-based therapeutic strategies.

## 1. Introduction

Cholelithiasis is defined as the development of solid crystalline calculi in the gallbladder or biliary tract, arising from abnormally elevated concentrations of cholesterol or bilirubin (a heme degradation product) in bile.^[1]^ It is a prevalent condition, occurring in roughly 10 to 20% of adults across the globe. More than 20% of individuals with cholelithiasis develop symptoms – such as biliary colic or biliary tract infections – at some point in adulthood. Clinically, cholelithiasis refers to the occurrence of symptoms or complications caused by gallstones within the gallbladder or bile ducts. From a diagnostic and therapeutic perspective, asymptomatic cholelithiasis are generally not considered cholelithiasis. Cholelithiasis is among the most economically burdensome gastrointestinal disorders worldwide.^[2]^

Cholecystic polyps are mucosal outgrowths that protrude into the lumen of the gallbladder, typically resulting from hyperplasia of the gallbladder epithelium.^[3]^ The underlying pathogenesis of these polyps remains largely unclear. In adults, the detection rate of cholecystic polyps by ultrasonography ranges from 1.5 to 4.5%, while their prevalence in cholecystectomy specimens varies between 0.004% and 13.8%.^[4–6]^ The human microbiota represents one of the most densely populated and rapidly evolving ecosystems in the body.^[7]^ Under normal physiological conditions, the microbiota and its metabolic products interact with the epithelial barrier and immune system to help maintain homeostasis.^[8]^ Bile is a physiological fluid synthesized and secreted by the liver and stored in the gallbladder. Traditionally, the biliary system has been considered sterile under healthy conditions.^[9]^ However, studies by Jiménez et al^[10]^ revealed the presence of bacteria in the bile and biliary mucosa of healthy pigs. Similarly, Molinero et al^[11]^ reported diverse bacterial taxa – such as Firmicutes, Bacteroidetes, Actinobacteria and Proteobacteria – in bile samples obtained from liver transplant donors without hepatobiliary disease. Although these donors were treated in intensive care units and their bile cannot be fully considered representative of that of healthy individuals, such findings nevertheless provide valuable evidence for the existence of a biliary microbiota.

However, most previous studies have primarily focused on the taxonomic composition of the biliary microbiota, while its functional characteristics and biological implications in biliary diseases remain poorly understood.^[12]^ Specifically, it is unclear how microbial functional potentials, such as metabolic and signaling pathways, differ between cholelithiasis and cholecystic polyps, and whether these differences contribute to disease development or reflect secondary responses to pathological conditions.

The term “metagenome” refers to the collective genetic material of all microorganisms present in a given environment.^[13]^ Advances in high-throughput sequencing technologies have revolutionized the identification and characterization of microbial species and functions, allowing for a comprehensive analysis using various bioinformatics tools.^[14]^ In this study, we extracted total genomic DNA from bile samples of patients with cholelithiasis and cholecystic polyps and performed metagenomic sequencing to characterize microbial diversity, community structure, and functional potential, aiming to elucidate the possible association between biliary microbiota dysbiosis and the pathogenesis of cholelithiasis and cholecystic polyps.

## 2. Methods

### 2.1. Study participants

This study was approved by the Second Affiliated Hospital of Zhejiang University School of Medicine. Bile samples were collected intraoperatively from 6 patients with cholelithiasis and 5 patients with cholecystic polyps. All participants were adults and provided written informed consent. Statistical analyses were performed using appropriate methods as described below, and a two-sided *P*-values < .05 was considered statistically significant. Sex distribution was assessed using Fisher exact test, and age was compared using the independent-samples Mann–Whitney *U* test. The 2 groups were matched and showed no significant differences in sex or age. Dietary habits were controlled during patient selection.

Inclusion criteria:

1. Age ≥ 18 years, with no sex restriction.
2. Patients with cholelithiasis diagnosed according to the guidelines of the Japanese Society of Gastroenterology,^[15]^ confirmed by transabdominal ultrasonography, with additional magnetic resonance cholangiopancreatography or CT imaging when necessary, and eligible for surgery.
3. Patients with cholecystic polyps detected by transabdominal ultrasonography and meeting surgical indications based on European guidelines^[16]^ with postoperative pathological confirmation.
4. All bile samples were collected intraoperatively.
5. No use of antibiotics, immunosuppressants, or microbiota-altering medications within 2 weeks prior to surgery.

Exclusion criteria:

1. Patients with systemic metabolic diseases (e.g., diabetes mellitus, hepatic or renal insufficiency, or immunodeficiency).
2. Use of antibiotics, immunosuppressants, or other microbiota-altering medications within 2 weeks prior to surgery.
3. Pregnant or lactating women.
4. History of major surgery or severe disease involving the biliary tract or gastrointestinal system.
5. Poor-quality samples (e.g., insufficient bile volume or contamination).
6. Acute cholecystitis was excluded on the basis of the Tokyo Guidelines.^[17]^

Sample collection and preservation:

1. All bile samples were aseptically collected during surgery under sterile conditions and placed into sterile containers.
2. Samples were maintained at 4°C and delivered to the laboratory within 2 hours of collection.

### 2.2. Metagenomic data acquisition

Bile samples were filtered through a sterile 0.22 µm membrane filter for the capture of microbial cells, after which the filter membranes were extensively washed with phosphate-buffered saline to eliminate remaining bile components. Microbial DNA was subsequently extracted from the filters using standard laboratory procedures.^[18]^ DNA quality was evaluated using 1% agarose gel electrophoresis. Genomic DNA was then sheared to an average fragment size of approximately 300 bp using the Covaris M220 system. A paired-end sequencing library was constructed using a TruSeq™ DNA Sample Prep Kit, which involved ligation of Y-shaped adapters to DNA fragments, removal of self-ligated fragments via magnetic bead purification, PCR amplification to enrich library templates, followed by denaturation using sodium hydroxide to produce single-stranded DNA.

### 2.3. Bridge PCR

For cluster generation, one end of each DNA fragment hybridized with primers immobilized on a flow cell, while the other end annealed to nearby primers to form a “bridge” PCR amplification was then performed to generate dense DNA clusters, which were subsequently linearized to single-stranded DNA. This process was carried out using the cBot TruSeq PE Cluster Kit v3-cBot-HS.

### 2.4. Sequencing

Sequencing was performed using a sequencing-by-synthesis approach with the TruSeq SBS Kit v3-HS (200 cycles). Modified DNA polymerase and 4 fluorescently labeled dNTPs were introduced, with the incorporation of a single base in each cycle. Fluorescence signals were detected by laser scanning to identify the incorporated nucleotides, after which the fluorescent and terminator groups were removed chemically to regenerate the 3′ end. This process was repeated in successive cycles until sequencing was completed (Table S1, Supplemental Digital Content 1).

### 2.5. Quality control of sequencing data

Raw sequencing reads were first evaluated using FastQC, followed by quality filtering and trimming with fastp.^[19]^ Clean reads were subsequently mapped to the human reference genome using Bowtie2^[20]^ to remove host-derived sequences, retaining high-quality non-host reads for downstream analyses.

### 2.6. Microbiome analysis

Microbial taxonomic annotation was performed using Kraken2,^[21]^ and species abundance was estimated with Bracken.^[22]^ Alpha diversity (Richness, Chao1, ACE, and Shannon indices) and beta diversity (Bray–Curtis and Jaccard indices) analyses were performed based on the generated abundance tables using the EasyMicrobiome^[23]^ pipeline. Differences in these diversity indices between the cholelithiasis and cholecystic polyp groups were evaluated using the Wilcoxon rank-sum test, with *P*-values < .05 considered statistically significant. Taxa with differential abundance among groups were identified via Linear discriminant analysis Effect Size (LEfSe) analysis, with multiple comparisons corrected using the Benjamini–Hochberg false discovery rate (FDR).

### 2.7. Functional enrichment analysis

The functional annotation of the metagenomic data was carried out using HUMAnN3,^[24]^ followed by Kyoto Encyclopedia of Genes and Genomes (KEGG) pathway enrichment analysis using the reporter score package to identify functional shifts potentially associated with disease conditions. In order to account for multiple comparisons and limit the FDR, Benjamini–Hochberg FDR correction was applied, enabling identification of functional shifts potentially associated with disease conditions.

## 3. Results

### 3.1. Demographic characteristics of the enrolled patients

**Figure 1.**
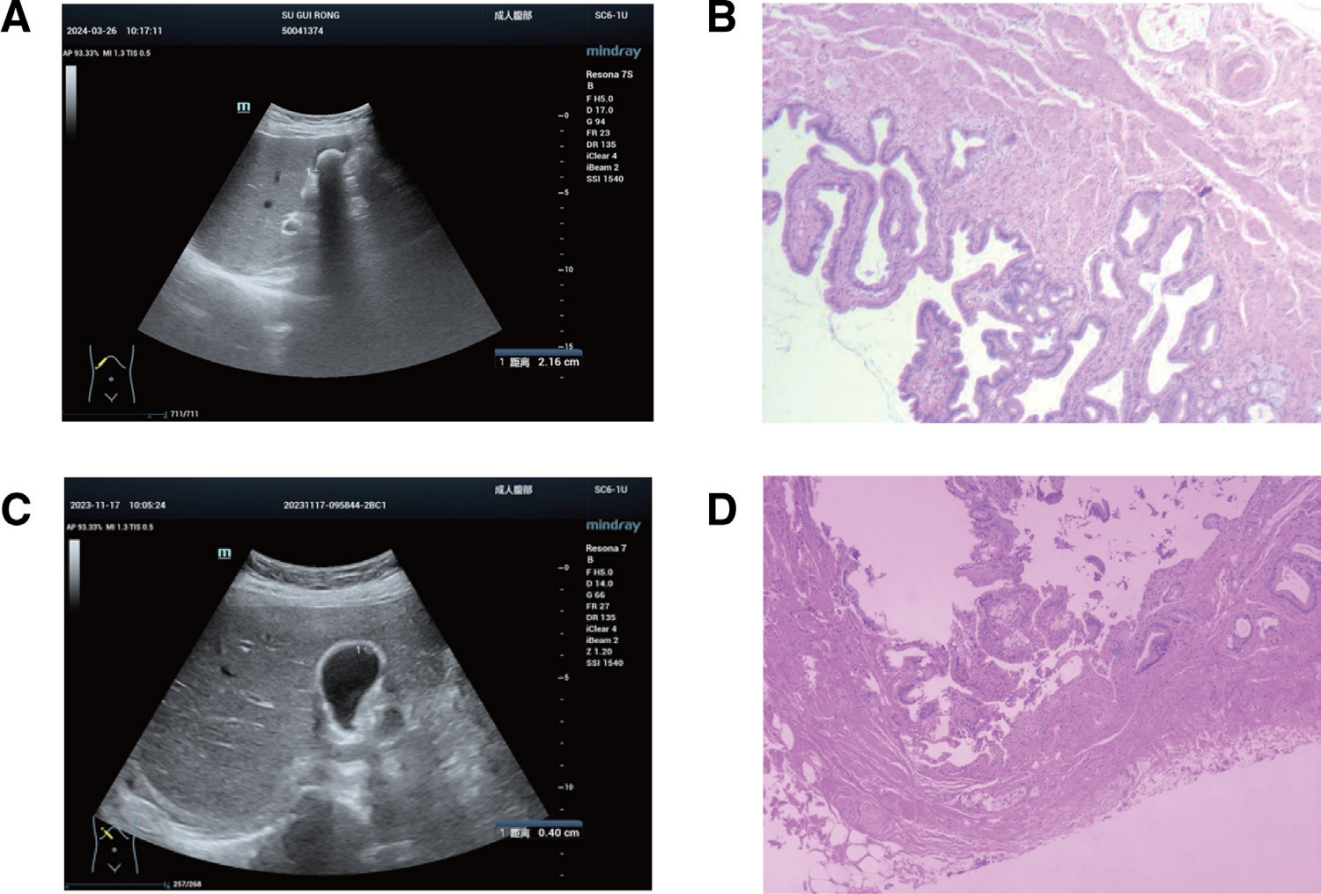
Ultrasound and pathological images of patients. (A) Ultrasound image of a patient with cholelithiasis; (B) Pathology image of a patient with cholelithiasis. (C) Ultrasound image of a patient with cholecystic polyps. (D) Pathology image of a patient with cholecystic polyps.

A total of 11 patients were included in this study, comprising 6 individuals with cholelithiasis and 5 with cholecystic polyps. All patients in the cholelithiasis group were diagnosed on the basis of a combination of abdominal ultrasound, magnetic resonance cholangiopancreatography, and histopathological examination (Fig. 1A–D). Similarly, patients in the cholecystic polyp group underwent the same diagnostic procedures for confirmation. Notably, all patients were also diagnosed with chronic cholecystitis on the basis of postoperative pathology.

A retrospective summary of patient demographics – such as age and sex – is presented in Table 1. Statistical analysis showed no significant differences in age or sex distribution between the cholelithiasis and cholecystic polyp groups.

**Table 1:** Basic patient information.

	Cholelithiasis	Cholecystic polyps	*P* value
Case number	6	5	NA
Age	54.4 ± 21.42	58.8 ± 11.23	.784
Sex	6 F/0 M	4 F/1 M	.455

### 3.2. Biliary microbiome profiling

**Figure 2.**
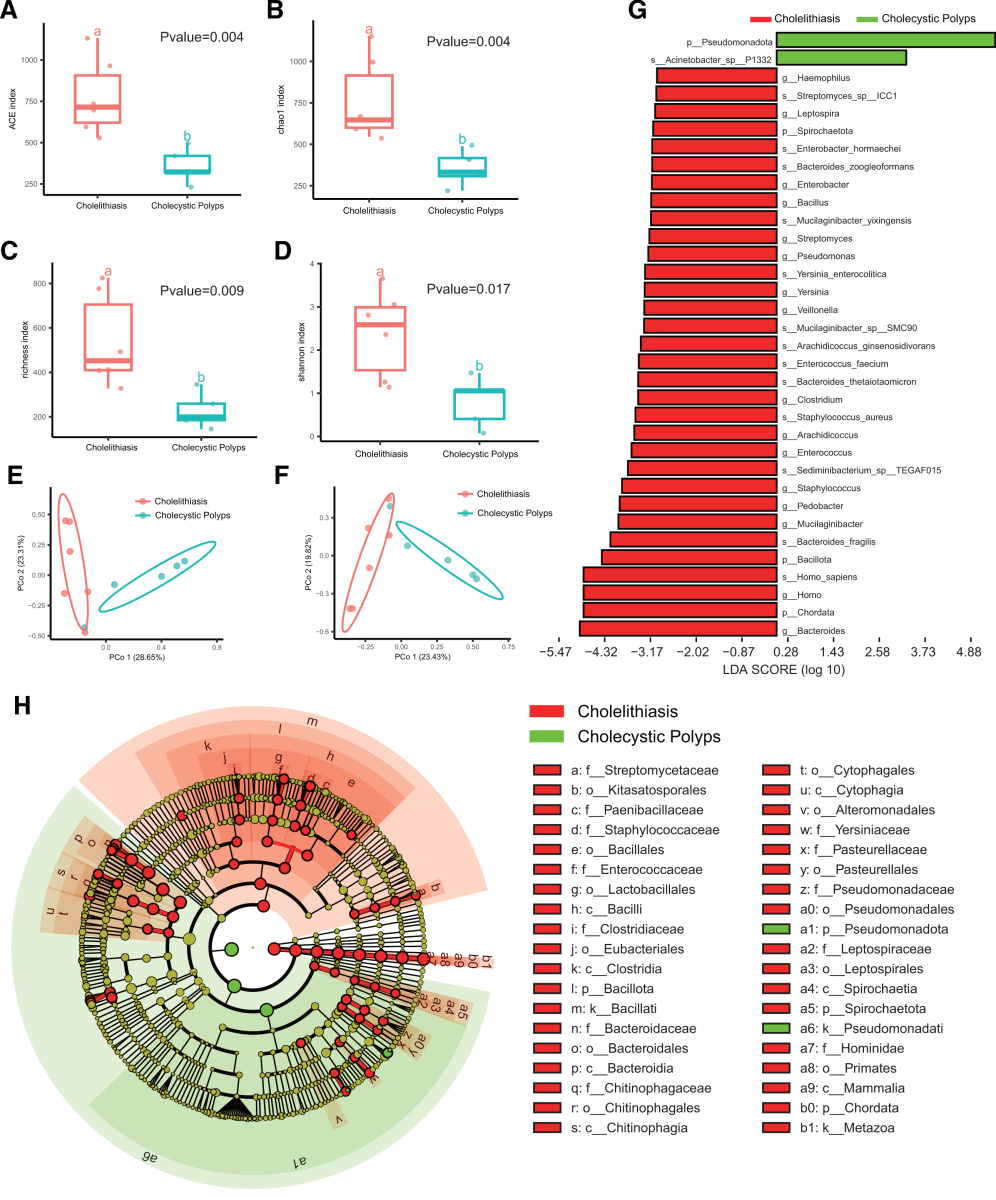
Differential analysis of biliary microbiota composition. (A) Alpha diversity was depicted through analysis of boxplots of the ACE index. (B) Alpha diversity was depicted through analysis of boxplots of the Chao1 index. (C) Alpha diversity was depicted through analysis of boxplots of Richness index. (D) Alpha diversity was depicted through analysis of boxplots of the Shannon index. (E) Beta diversity was visualized using principal coordinate analysis based on weighted Bray–Curtis distances. (F) Beta diversity was visualized using principal coordinate analysis based on weighted Jaccard distances. (G) LEfSe cladogram; (H) LDA score of differential features between cholelithiasis and cholecystic polyp microbiota. LDA = Linear discriminant analysis, LEfSe = Linear discriminant analysis Effect Size.

The diversity of the bile microbiota at the genus level was assessed both within groups (α-diversity) and between groups (β-diversity). Several indices were calculated to evaluate α-diversity: Richness, which represents the actual number of observed species in a sample, provides the most direct measure of community composition (Fig. 2A); Chao1 is a non-parametric estimator that predicts the abundance of rare or unobserved species (Fig. 2B); observed species estimate the number of unique operational taxonomic units identified per sample; and ACE is another non-parametric richness estimator, similar to Chao1 (Fig. 2C). The Shannon index accounts for both species richness and evenness, providing a composite measure of microbial diversity (Fig. 2D). Patients with cholelithiasis exhibited significantly higher bile microbial diversity than those with cholecystic polyps, as reflected by increased Richness (*P* = .009), Chao1 (*P* = .004), ACE (*P* = .004), and Shannon indices (*P* = .017).

**Figure 3.**
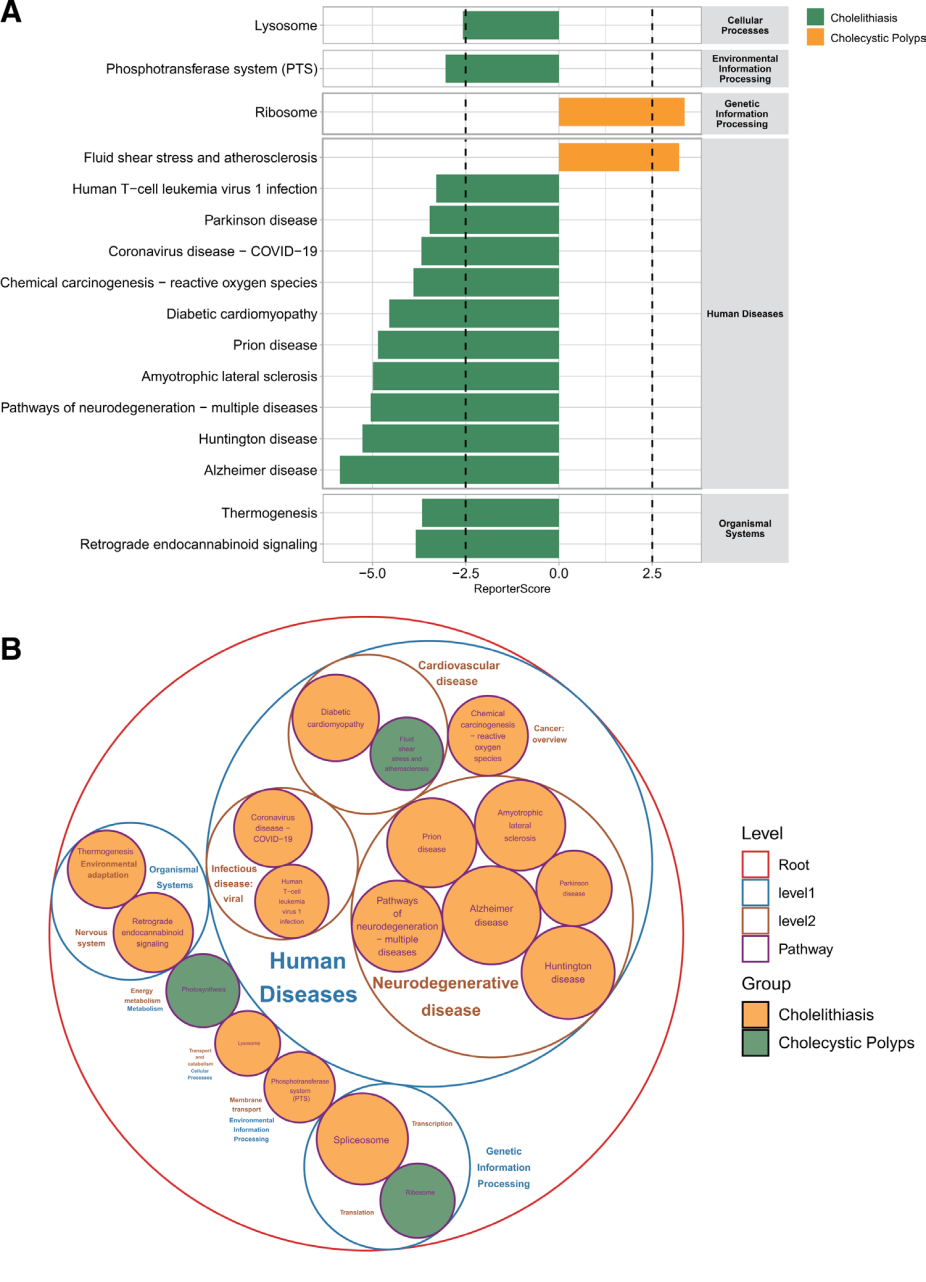
Kyoto Encyclopedia of Genes and Genomes (KEGG) pathway enrichment analysis. (A) Reporter score plot showing enriched KEGG pathways in the biliary microbiota of cholelithiasis (green) and cholecystic polyp (orange) patients. (B) Circle packing plot illustrating hierarchical clustering of enriched KEGG pathways by functional category.

To assess β diversity, we calculated the Bray–Curtis and Jaccard distances between the 2 groups and conducted principal coordinate analysis on the basis of these metrics. The results revealed significant differences in bile microbial community structure between the cholelithiasis and cholecystic polyp groups (Fig. 3A). Overall, these findings indicate notable differences in microbial composition between the 2 patient groups, with significantly higher microbial diversity in the cholelithiasis group, suggesting that their biliary microbiota may be more strongly influenced by disease status.

To determine taxa with differential abundance between the 2 groups, LEfSe analysis was performed using a signed Linear discriminant analysis score threshold of >3 or < −3. At the phylum level, the cholecystic polyp group was enriched only in Pseudomonadota, whereas the cholelithiasis group showed specific enrichment of Mycoplasmatota, Planctomycetota, Campylobacterota, Fusobacteriota, Thermodesulfobacteriota, Spirochaetota, and Bacillota. At the genus level, no specific genera were enriched in the cholecystic polyp group, whereas 35 genera – including *Bacteroides*, *Mucilaginibacter*, *Pedobacter*, and others – were significantly enriched in the cholelithiasis group. These findings are consistent with those of the α diversity analysis.

### 3.3. KEGG pathway enrichment analysis

KEGG pathway enrichment analysis of the biliary microbiota revealed that the genes in the cholelithiasis group (patients with cholelithiasis) were enriched in multiple disease-related pathways. These included pathways associated with neurodegenerative diseases (such as Parkinson disease and Alzheimer disease), cardiovascular conditions (such as atherosclerosis and diabetic cardiomyopathy), viral infections (including COVID-19 and HTLV-1), and chemically induced carcinogenesis driven by oxidative stress. Notably, enrichment was predominantly observed in neurodegenerative disease pathways, suggesting a potential link between cholelithiasis and increased susceptibility to such disorders. In contrast, the cholecystic polyp group (patients with cholecystic polyps) was enriched in biosynthetic pathways (e.g., ribosome-related) and fluid shear stress pathways, which may indicate a microbiota-mediated protective effect against atherosclerosis. Overall, these findings suggest that dysbiosis of the biliary microbiota has a greater effect on patients with cholelithiasis.

## 4. Discussion

Traditionally, the biliary tract in healthy individuals has been considered sterile. However, recent studies suggest that microbes may exist in the bile of healthy individuals, although there is no definitive evidence to confirm this. In contrast, the presence of microorganisms in the bile of patients with biliary diseases has been clearly established. Previous research has demonstrated that the biliary microbiota is closely associated with the onset and progression of biliary diseases. For instance, Li et al^[25]^ reported dynamic changes in the composition of the biliary microbiota during disease progression, with distinct microbial profiles observed in different types of biliary diseases. Disruption of the biliary microbial balance may lead to inflammation and gallstone formation, potentially contributing to the development of cholangiocarcinoma. Therefore, understanding the relationship between the biliary microbiota and biliary diseases is essential for elucidating disease pathogenesis and developing new therapeutic approaches.

In this study, we reported that the microbial diversity in the bile of cholelithiasis patients was significantly greater than that in the bile of patients with cholecystic polyps. These findings may reflect differences in microbial involvement across distinct biliary pathologies. Notably, enrichment of the phylum Pseudomonadota was observed in the cholecystic polyp group, which is consistent with the findings of Avilez-Jimenez et al,^[26]^ who reported similar enrichment in cholangiocarcinoma patients in Mexico. Given that cholecystic polyps are benign gallbladder tumors, the presence of Pseudomonadota under both conditions suggests that this phylum may play a critical role in polyp and cancer development and could serve as a potential biomarker for cholecystic polyps.

Gallstones can be classified into cholesterol stones, pigment stones, and mixed types. In the case of pigment stones, bacteria capable of producing hydrolytic enzymes, such as *Klebsiella* and *Enterococcus*, may contribute to stone formation through biofilm formation. Peng et al^[27]^ reported that *Pseudomonas* was among the predominant genera in both bile and cholesterol stones in cholelithiasis patients. Swidsinski et al^[28]^ also detected bacterial DNA from *Propionibacterium*, *Clostridium*, and *Enterobacter* in cholesterol-rich mixed stones. In our study, we also observed enrichment of *Enterobacter*, *Clostridium*, and *Pseudomonas* in the bile of patients with cholelithiasis, which is consistent with findings from Peng and Swidsinski. These findings support the hypothesis that these genera may be involved in the pathogenesis of cholelithiasis. Furthermore, we identified 32 other genera that are not commonly reported in biliary microbiota studies, including *Arachidicoccus*, *Staphylococcus*, *Pedobacter*, *Mucilaginibacter*, *Bacteroides*, and others. The presence of these genera may indicate a shift in the biliary microenvironment during dysbiosis that favors gallstone formation over polyp development. Collectively, these results suggest that the microbial contribution to disease development may be more prominent in patients with cholelithiasis than in those with cholecystic polyps.

KEGG pathway enrichment analysis revealed that the biliary microbiota in cholelithiasis patients was significantly enriched in multiple human disease-associated pathways. Although bacteria do not contain human genes, they share conserved biological processes with the host, and homologous genes can be mapped to human disease pathways in KEGG. In our analysis, neurodegenerative diseases, such as Alzheimer disease, Parkinson disease, and Huntington disease, represented the most enriched category. This could be related to the elevated cholesterol levels commonly observed in patients with cholelithiasis, which have been implicated in the pathogenesis of various neurodegenerative disorders.^[29]^ These findings indicate that the functional signatures of the biliary microbiota in cholelithiasis may exhibit homology with pathways implicated in neurodegenerative diseases. Such associations likely reflect shared metabolic or inflammatory mechanisms rather than a direct causal relationship. In contrast, the cholecystic polyp group (cholecystic polyps) was enriched in ribosome-related pathways and fluid shear stress, possibly indicating a microbiota-mediated protective effect against atherosclerosis. Importantly, these interpretations are exploratory and hypothesis-generating in nature, as they are derived from predictive bioinformatic analyses rather than direct mechanistic evidence.

We also attempted to apply metagenomic assembly and binning strategies to obtain high-resolution taxonomic and functional insights from bile samples. However, metagenomic assembly yielded limited data, and binning was unsuccessful. This may be attributed to the inherently low microbial biomass in bile, combined with insufficient sequencing depth and a high proportion of host (human) DNA contamination. These factors dilute microbial signals and hinder effective assembly and binning. Although metagenomic assembly and binning are powerful tools for exploring complex microbial communities, their application in bile samples remains technically challenging.

In summary, in this study, the taxonomic composition and functional profiles of biliary microbiota were compared between patients with cholelithiasis and those with cholecystic polyps, revealing the association between microbial dysbiosis and biliary disease progression. Notably, our data suggest that patients with cholelithiasis may have a greater susceptibility to neurodegenerative diseases, although direct evidence is still lacking. At the same time, this study has several limitations, primarily related to the relatively small sample size. With only 11 bile samples analyzed in this small cohort, the statistical power to detect subtle microbial differences was limited, and the findings may have been influenced by inter-individual variability. The small cohort also constrains the generalizability of the results to broader patient populations. Moreover, the absence of a healthy control group restricts the interpretation of disease-associated microbial alterations. Because bile samples from healthy individuals are difficult to obtain for ethical and technical reasons, it remains uncertain whether the observed microbial differences represent disease-specific changes or deviations from the normal biliary microbiota. While metagenomic sequencing has enhanced our understanding of the biliary microbiota, its functional complexity and ecological dynamics remain to be fully elucidated. Future studies with larger cohorts, the integration of comprehensive public health data, and deeper biochemical investigations will be essential to validate these findings and uncover underlying mechanisms.

## Acknowledgments

The authors would like to thank the staffs in The Second Affiliated Hospital Zhejiang University School of Medicine and the staffs in College of Pharmaceutical Science & Collaborative Innovation Center of Yangtze River Delta Region Green Pharmaceuticals, Zhejiang University of Technology. And we thank everyone who has critical reading and editing on this article. We also gratefully acknowledge platform support from the Zhejiang Key Laboratory of Green, Low-carbon and Efficient Development of Marine Fishery Resources.

## Author contributions

**Conceptualization:** Yuanquan Yu, Chenjie Wang.

**Data curation:** Yuanquan Yu, Chenjie Wang, Xiang Pan, Chao Ding.

**Formal analysis:** Yuanquan Yu, Chenjie Wang, Xiang Pan, Chao Ding.

**Writing – original draft:** Yuanquan Yu, Chenjie Wang, Jianwei Chen.

**Writing – review & editing:** Yuanquan Yu, Chenjie Wang, Jianwei Chen.


