# Supplementary material for: Metagenomic profiling of biliary microbiota reveals distinct microbial and functional features in cholelithiasis and cholecystic polyps
Source: Medicine (Baltimore). 2026 Jun 12;105(24):e49251. doi: 10.1097/MD.0000000000049251 (PMC13268569; doi:10.1097/MD.0000000000049251)
Supplement: Supplementary file 1 [file medi-105-e49251-s001.docx]

Table S1. Sample IDs

| Accession | Group | SPUID |
| --- | --- | --- |
| SAMN50315042 | Cholecystic Polyps | bile metagenome |
| SAMN50315043 | Cholelithiasis | bile metagenome |
| SAMN50315044 | Cholecystic Polyps | bile metagenome |
| SAMN50315045 | Cholelithiasis | bile metagenome |
| SAMN50315046 | Cholelithiasis | bile metagenome |
| SAMN50315047 | Cholelithiasis | bile metagenome |
| SAMN50315048 | Cholecystic Polyps | bile metagenome |
| SAMN50315049 | Cholelithiasis | bile metagenome |
| SAMN50315050 | Cholelithiasis | bile metagenome |
| SAMN50315051 | Cholecystic Polyps | bile metagenome |
| SAMN50315052 | Cholecystic Polyps | bile metagenome |
